# Progressive adaptation of whole-limb kinematics after peripheral nerve injury

**DOI:** 10.1242/bio.028852

**Published:** 2018-08-15

**Authors:** Young-Hui Chang, Stephen N. Housley, Kerry S. Hart, Paul Nardelli, Richard T. Nichols, Huub Maas, Timothy C. Cope

**Affiliations:** 1 Department of Neuroscience, Cell Biology and Physiology, Boonshoft School of Medicine, Wright State University, Dayton, Ohio 45435, USA; 2 School of Biological Sciences, Georgia Institute of Technology, Atlanta, Georgia 30332, USA; 3 The Coulter Department of Biomedical Engineering Georgia Tech College of Engineering and Emory School of Medicine Georgia Institute of Technology, Atlanta, Georgia 30332, USA; 4 Department of Human Movement Sciences, Faculty of Behavioural and Movement Sciences, Vrije Universiteit Amsterdam, Amsterdam Movement Sciences, 1081 HV Amsterdam, Netherlands

**Keywords:** Functional recovery, Locomotor compensation, Muscle paralysis

## Abstract

The ability to recover purposeful movement soon after debilitating neuromuscular injury is essential to animal survival. Various neural and mechanical mechanisms exist to preserve whole-limb kinematics despite exhibiting long-term deficits of individual joints following peripheral nerve injury. However, it is unclear whether functionally relevant whole-limb movement is acutely conserved following injury. Therefore, the objective of this longitudinal study of the injury response from four individual cats was to test the hypothesis that whole-limb length is conserved following localized nerve injury of ankle extensors in cats with intact nervous systems. The primary finding of our study was that whole-limb kinematics during walking was not immediately preserved following peripheral nerve injuries that paralyzed subsets of ankle extensor muscles. Instead, whole-limb kinematics recovered gradually over multiple weeks, despite having the mechanical capacity of injury-spared muscles across all joints to achieve immediate functional recovery. The time taken to achieve complete recovery of whole-limb kinematics is consistent with an underlying process that relies on neuromuscular adaptation. Importantly, the gradual recovery of ankle joint kinematics remained incomplete, discontinuing once whole-limb kinematics had fully recovered. These findings support the hypothesis that a whole-limb representation of healthy limb function guides a locomotor compensation strategy after neuromuscular injury that arrests progressive changes in the joint kinematics once whole-limb kinematics is regained.

## Abstract (precis)

**Summary:** Longitudinal study of four cats documented recovery of locomotor kinematics in one hindlimb following nerve injury and muscle paralysis focused at the ankle joint. Whole-limb length was not conserved immediately, but recovered pre-injury levels gradually through kinematic adjustments distributed across joints.

## INTRODUCTION

The ability to recover purposeful movement soon after debilitating neuromuscular injury is essential to animal survival. Various mechanisms exist to counteract impairments and conserve functionally relevant movement (Donelan and Pearson, 2004; Frigon and Rossignol, 2007; Gritsenko et al., 2001; Prilutsky et al., 1996). For example, muscles that lose innervation following peripheral nerve injury are initially paralyzed, but can regain function through nerve regeneration. Restoration through nerve regeneration, however, is imperfect (Alvarez et al., 2010; Haftel et al., 2005), and it can require many weeks to months to return meaningful function to the reinnervated muscles (Fu and Gordon, 1997; Gordon and Stein, 1982; Gregor et al., 2018; Navarro et al., 2007). Functional recovery may also be achieved by progressive muscle hypertrophy after denervation of its synergists (Walsh et al., 1978), although this process may require 10 or more days to develop (Degens et al., 1995). Alternatively, more rapid functional recovery of movement can be achieved through neural adaptations such as modification of sensory feedback (Donelan and Pearson, 2004), reflex pathways (Frigon and Rossignol, 2007), or spinal circuits (Gritsenko et al., 2001) that adjust activation of injury-spared muscles (Gregor et al., 2018; Maas et al., 2010; Prilutsky et al., 2011).

Despite the presence of various mechanisms to compensate for injury by exploiting biomechanical redundancy (Bunderson et al., 2008), it is unknown how these time-dependent processes at the joint level may interact and influence recovery of functional whole-limb movement (Degens et al., 1995; Donelan and Pearson, 2004; Frigon and Rossignol, 2007; Pearson et al., 1999). As a result, global parameters of movement such as whole-limb length and orientation that may be preferentially encoded in the central nervous system (Bosco and Poppele, 2000, 2003; Bosco et al., 2000) have recently come into focus following isolated nerve injury (Bauman and Chang, 2013; Chang et al., 2009; Maas et al., 2007).

Within 1 month after experimental denervation of a subset of ankle extensor muscles, cats and rats exhibit normal whole-limb length and orientation of the injured limb during walking, despite lingering kinematic irregularities at each of the joints (Bauman and Chang, 2013; Chang et al., 2009). It is possible that whole-limb length reaches normal values as early as 1 week after nerve injury as reported by Maas et al. (2007), but this time point is uncertain, because these data were pooled over the first 7 weeks after denervation. The possibility for immediate recovery of whole-limb kinematics has been examined, but only in cats in which descending motor commands have been experimentally altered. Under conditions of acute decerebration (Stahl and Nichols, 2011) or chronic spinalization (Bouyer et al., 2001), the animal does not immediately conserve normal whole-limb kinematics. All considered, the early stages of whole-limb response to partial denervation of ankle extensors remains unknown for conscious animals with intact central nervous systems.

Clarifying the early development of kinematic compensation in awake, behaving cats should assist in identifying the underlying neuromuscular compensatory mechanisms. The finding that whole-limb kinematics is corrected immediately after denervating a subset of the primary muscles acting at a joint would indicate that existing neural and/or mechanical mechanisms can compensate to maintain whole-limb kinematics. Alternatively, whole-limb kinematics might not be immediately restored, suggesting that the underlying neuromuscular compensatory mechanisms involve processes of learning or plasticity that develop gradually. Thus, by knowing the early development of whole-limb recovery from injury, we might gain better understanding of the processes that enable animals to achieve purposeful and robust locomotion well in advance of time-consuming tissue regeneration.

Therefore, the objective of the present study was to test the hypothesis that whole-limb length is conserved immediately following localized nerve injury of ankle extensors in cats with intact nervous systems. The kinematics of voluntary locomotion were examined in a convenience sampling of cats before and after unilateral denervation of one or another subset of the primary ankle extensor muscles that were utilized in other electrophysiological studies. Despite the variety of specific nerves injured, we were able to test the general hypothesis about limb-level versus joint-level kinematic changes after a localized injury. Results were uniform across individual cats, showing that whole-limb length was not conserved in the first day post-injury. Instead, limb length deviated considerably from pre-injury values for more than one week in every animal. Because recovery of whole-limb kinematics was not immediate, it seemed to rely on the development of a new motor strategy that falls outside the normal capacity of existing locomotor compensation mechanisms. We also found that recovery of whole-limb kinematics progressed gradually over a week or longer in parallel with recovery of ankle kinematics. Importantly, once whole-limb kinematics reached pre-injury values, ankle joint kinematics ceased to recover before reaching its pre-injury values. These findings support the hypothesis for a neuromuscular compensation strategy that prioritizes task-level function of the whole-limb over individual joints (Chang et al., 2009).

## RESULTS

### Whole-limb and joint kinematics day 1 after nerve injury

**Fig. 1.**
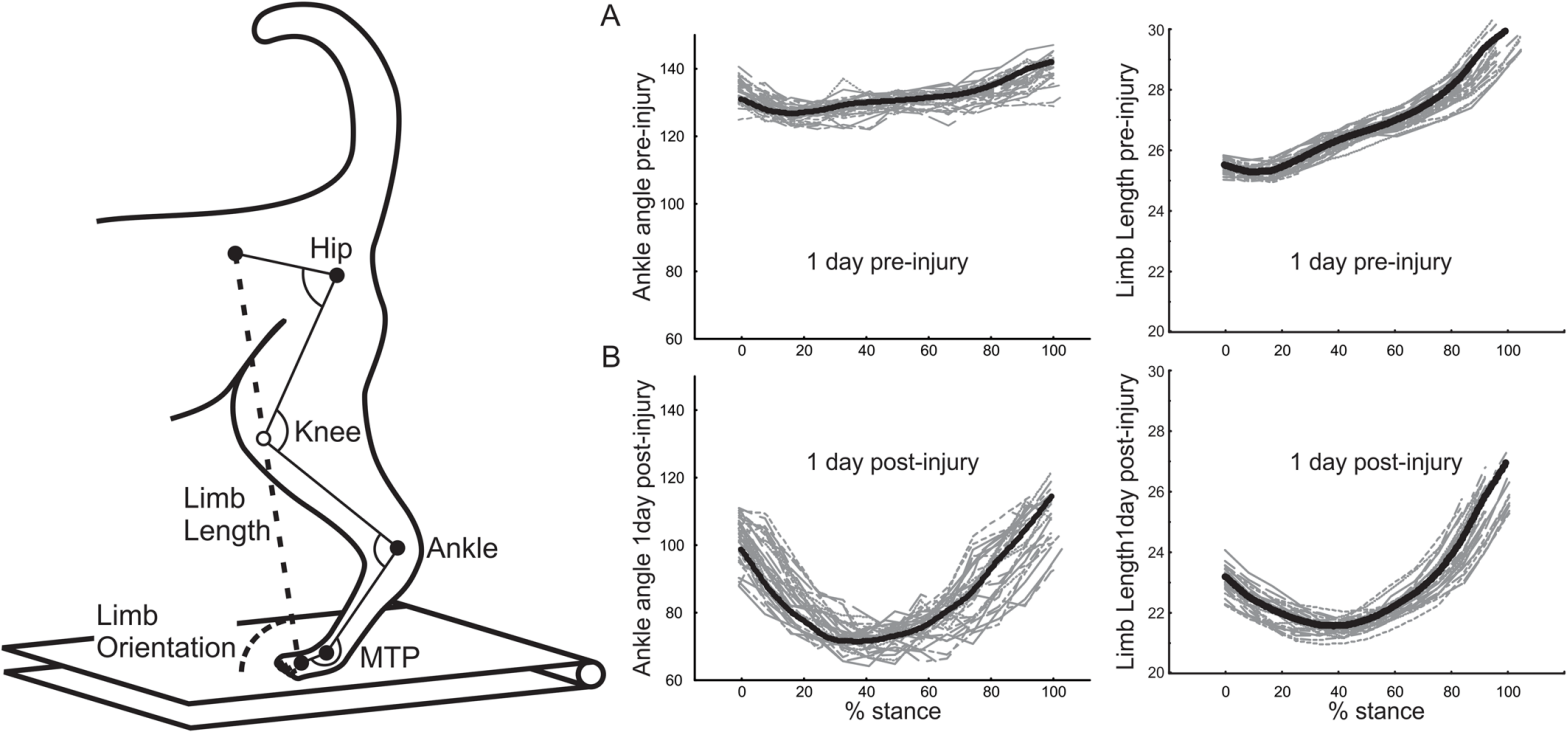
**Whole-limb kinematics prior to and days after partial limb paralysis.** Diagram of cat left hind limb, identifying limb segments and angles used in analyzing kinematics during treadmill walking (∼0.7 m/s). Markers (filled circles) were taped to shaved skin overlying five boney landmarks (proximal to distal): iliac crest, greater trochanter, lateral epicondyle, lateral malleolus, fifth metatarsal head and fifth distal phalanx. Landmarks and their linked segments (interconnecting lines) were tracked in two-dimensional space and used to calculate included angles (joint kinematics) at the hip, knee and ankle joints and for the excluded angle for the metatarsal-phalangeal (MTP) joint. Limb length and orientation (whole-limb kinematics) were measured, respectively, from dashed line segment joining iliac crest to distal phalanx; and its angle with treadmill surface. (A,B) Trajectories of selected kinematic parameters during treadmill walking before and after injury (partial denervation of ankle extensors). Trajectories are shown for ankle-joint angle (left column) and for whole-limb length (right column), recorded from one cat during the stance phase of steps taken one day before (top plots) and one day after (middle plots) nerve injury (xLGSP). Abscissa is the percent of stance phase between paw contact (0%) and removal (100%) from treadmill surface. Mean trajectories (thick lines) were obtained from 20-30 individual steps (thin lines) before and after injury.

**Fig. 2.**
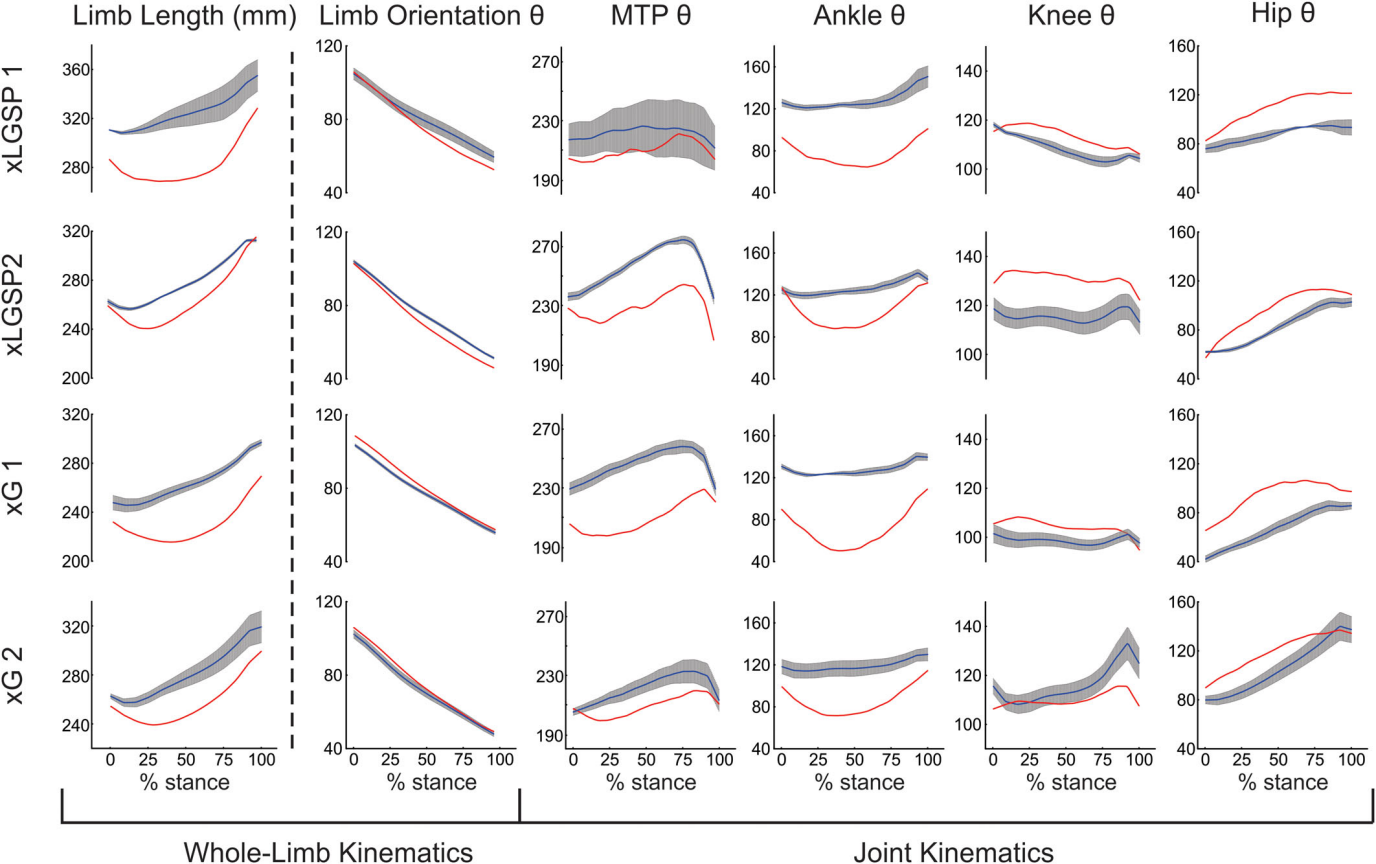
**Kinematic changes within 1 day after partial limb paralysis.** Trajectories were averaged over the stance phase of multiple steps during treadmill walking for joint angles (θ in degrees) at MTP, ankle, knee and hip joints; and for limb orientation angle and length. Nerves in the left hindlimb supplying LGSP muscles were severed in two cats (xLGSP1 and xLGSP2), and those supplying G muscles were severed in two other cats (xG1 and xG2). Mean stance-phase trajectories are shown for steps taken in the week before nerve section (blue lines and grey shading showing, respectively, mean±1 s.d. for steps measured over three separate days) and for steps taken between 12-20 h after nerve transection (red lines).

Changes in kinematic parameters were evident throughout the injured hindlimb in the first 20 h after injury. Data presented in Figs 1 and 2 were obtained from the first sessions of continuous walking at constant treadmill speed (0.7 m/s). The very first steps taken on the treadmill (<15 steps) after surgery were highly inconsistent and, therefore, were excluded from analysis.

### Whole-limb kinematics

Whole-limb length for each of the four; was clearly shorter over virtually the entire stance phase of gait when compared with before and within the first 20 h after nerve injury, limb length was clearly shorter over virtually the entire stance phase for all four cats (Figs 1 and 2). Before nerve injury, the limb length trajectory in each cat exhibited slight and short-lasting decreases in limb length (yield) early in the stance phase (≤15% at the beginning of stance) followed by a relatively smooth increase in length throughout the remainder of stance. On the first day after nerve injury, the yield in stance phase limb length was greater in magnitude and duration than before injury (Fig. 2). Differences between cats were not in any apparent way related to injury type, i.e. to xG versus xLGSP. These findings establish that whole-limb length was not immediately conserved by cats performing a volitional locomotor task. The decline in whole-limb length was similar in magnitude to that measured in the acutely decerebrated cat immediately upon denervating selected ankle extensor muscles (Stahl and Nichols, 2011).

On the first day after injury, the trajectory of whole-limb orientation deviated slightly from the pre-injury trajectory (Fig. 2). A pattern emerged in relation to the type of injury, with limb orientation increasing in both xG cats, but decreasing in the two xLGSP cats.

### Joint kinematics

Fig. 2 illustrates kinematic changes in individual joints of the injured hindlimbs. The excessive ankle flexion observed throughout stance was the expected consequence of denervating several of the primary ankle extensors. Exaggerated ankle flexion (Fig. 2) seems a major contributor to the decline in whole-limb length. The excluded angle at the MTP joint also decreased, probably as a direct biomechanical consequence of the large ankle yield.

The remaining two joint angles measured in this study, i.e. hip and knee joint angles, generally demonstrated increased stance-phase extension on the first day after nerve injury. Fig. 2 shows changes toward greater extension (or less flexion) at the hip and knee that exceeded one standard deviation of the pre-injury mean across most of their stance phase trajectories. Increased extension at the hip and knee were in the direction of restoring whole-limb length in partial compensation for increased ankle flexion, and these increases occurred within 20 h of injury.

### Limb and joint kinematics in week 1 after nerve injury

**Fig. 3.**
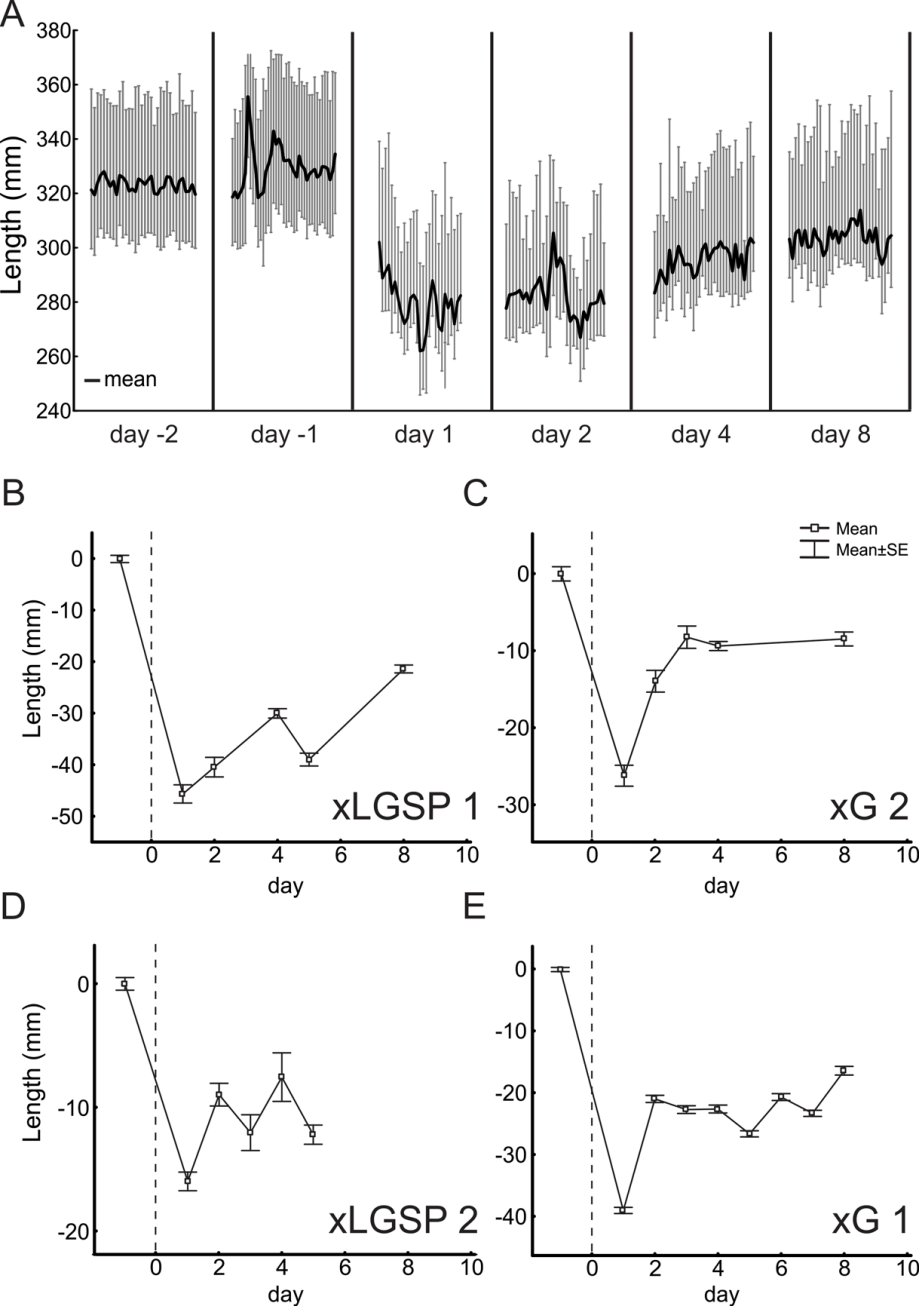
**Limb length shortened for several days post-injury.** (A) Full range in limb length for stance phase of successive steps (vertical grey lines) and its average value (dark line) measured in cat xLGSP1 before injury (days 2 and 1) and at intervals after injury. (B-E) Plots of average daily limb length (x±s.d.) for all steps (20-30) for each of four cats; vertical dotted lines signify the day of nerve injury (day 0); horizontal dashed lines delimit 95% confidence intervals obtained from steps taken over at least 3 days in the week before nerve injury.

The deficit in whole-limb length that was evident on post-injury day 1 extended several days longer as shown in Fig. 3. Reduction in whole-limb length during stance was evident whether measured as minimum, maximum, or average length (Fig. 3A). A return toward pre-injury limb length was evident (Fig. 3B-E), but incomplete in the first week post-injury for all cats.

### Recovery of whole-limb kinematics and lingering joint deficits over the first three weeks after injury

**Fig. 4.**
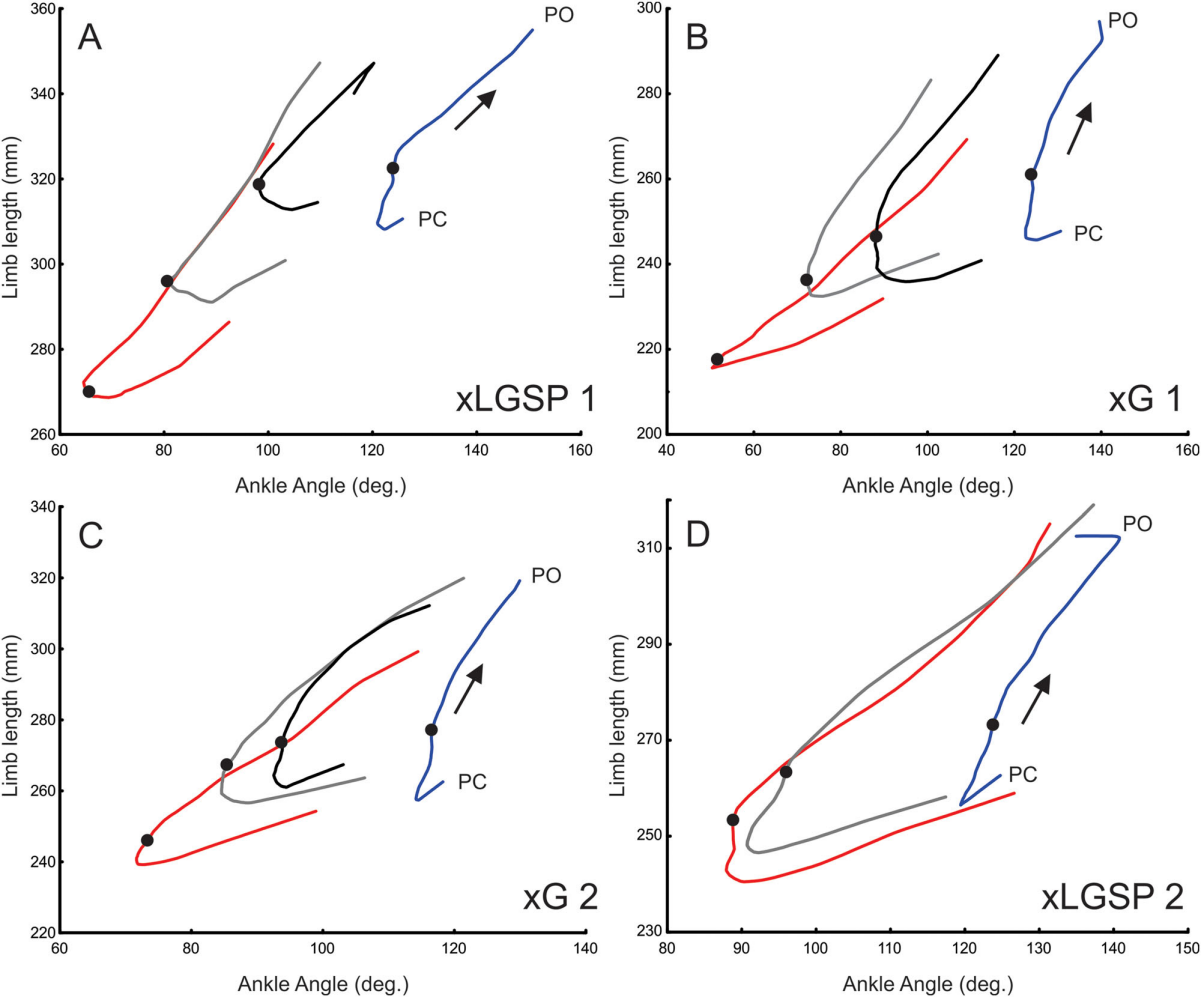
**Coordinated recovery of whole-limb and joint level kinematics during partial paralysis of ankle extensor muscles.** (A-D) Plots of limb length versus ankle angle measured for the stance phase of steps progressing (arrows) from PC through mid-stance (black dots) to PO the treadmill surface during walking. Trajectories were averaged over multiple steps recorded before nerve injury (blue lines) and after injury (red line, day 1; grey line, week 1; black line, week 3). Note that xLGSP2 was terminated before week 3).

Fig. 4 illustrates the relationship between whole-limb length and ankle angle as they change together during the stance phase of steps averaged before injury and at 1 day, 1 week and 3 weeks after injury. A prominent feature of steps taken after injury, particularly on the first day, was a concomitant decline in whole-limb length and ankle angle during the first half of the stance phase. Over the first 3 weeks post-injury, the relation between limb length and ankle joint angle at mid-stance progressed toward its pre-injury values (Fig. 4A-D). Over this same time period, no substantial changes in stride length or stance time were found between time points within cats and, thus, changes in stride parameters did not bias these other results (data not shown).

Even at 3 weeks, however, ankle joint kinematics failed to return to the pre-injury range in contrast to whole-limb length, which recovered fully. Recovery of ankle angle, although correlated with whole-limb length, was distinguished by its failure to attain pre-injury levels. The curves in Fig. 4 indicate length-angle relations returned into the pre-injury range on the limb length (y) axis, whereas it did not reach the pre-injury range on the ankle joint angle (x) axis (Fig. 4A-D). These comparisons corroborate preferential recovery of whole-limb length over ankle and other joint angles (Boeltz et al., 2013; Chang et al., 2009), and establish its emergence between 1 and 3 weeks after injury.

## DISCUSSION

The present study is the first to show a failure to preserve whole-limb kinematics during walking in the first few days following peripheral nerve injuries that paralyzed subsets of ankle extensor muscles. Earlier reports on adult cats with similar injuries report that whole-limb length recovered fully by 1 month, despite the persistence of abnormal joint kinematics (Abelew et al., 2000; Chang et al., 2009; Maas et al., 2007). While the underlying mechanism remains unknown, determining the onset of whole-limb recovery yields insights described below.

Since there is no possibility for recovery of the denervated muscles, the gradual changes in ankle joint kinematics in the first few weeks following injury must be due to changes in the relative activations and mechanical contributions from the remaining intact ankle extensor muscles. As we only tested cats walking on a level surface at a moderate speed (0.7 m/s), we can assume that the intact muscles were well within their maximal force-producing capacity to restore normal joint and limb kinematics immediately after paralyzing a subset of ankle extensors. Prilutsky and colleagues (Prilutsky et al., 1996) showed ankle extensors in the cat have the capacity to nearly double their positive work output relative to walking at a similar moderate speed (0.8 m/s). After denervation of LG and S, EMG activity of the intact MG muscle is found to be higher during upslope walking than during level walking (Maas et al., 2010), further indicating that the contribution by the intact synergist muscles was not limited in their ability to increase activation.

### Whole-limb kinematics recover gradually

Despite the muscular capacity for immediate restoration (Prilutsky et al., 1996) recovery of whole-limb length was not immediate, but instead occurred gradually over weeks following limited nerve injury. Moreover, recovery of limb kinematics was not accelerated by maintaining complete supraspinal access to spinal sensorimotor systems in cats having an intact and undamaged central nervous system. These findings raise the question: what processes are responsible for delaying whole-limb recovery? Some insight is offered by our finding that limb length recovery tracked kinematic recovery of its component ankle joint, for which some processes underlying recovery have been determined (Pearson et al., 1999; Rossignol and Bouyer, 2004). At the ankle, kinematic recovery depends on limb use, as evidenced by the observation that gradual recovery is postponed by splinting the partially paralyzed limb and is not initiated until the splint is removed and mobilization is restored (Pearson et al., 1999). Dependence on time and limb usage suggests some sort of underlying neural or mechanical adaptation, i.e. plasticity or learning.

Neural or mechanical adaptation is inferred as the underlying basis for the recovery of ankle kinematics that occurs slowly, but well in advance of any significant peripheral reinnervation of denervated (i.e. paralyzed) ankle extensors. Progressive recovery of ankle kinematics involves increased activation of injury-spared ankle extensor muscles (Fouad and Pearson, 1997; Frigon and Rossignol, 2007; Pearson et al., 1999; Whelan et al., 1995). These adaptations are shown to require feedback from large-diameter sensory afferents (Pearson et al., 2003), and they can be mediated by the spinal cord in the absence of supraspinal input (Bouyer et al., 2001; Takeoka et al., 2014). Although the exact sites of adaptation are undefined and are outside the scope of this longitudinal report of four individual cats, progressive alterations are found in pre-motor reflex circuits (Donelan and Pearson, 2004; Fouad and Pearson, 1997; Frigon, 2011; Whelan, 1996) and/or other sources of spinal and supraspinal synaptic drive to motoneuron pools (Bouyer et al., 2001; Fouad and Pearson, 1997; Frigon and Rossignol, 2009; Gritsenko et al., 2001). In addition, muscular and/or connective tissue plasticity may contribute to the kinematics results, especially the later time point (>10 days) (Degens et al., 1995; Walsh et al., 1978; Whelan and Pearson, 1997). Taken together, we suggest that these processes are responsible for setting the rate of recovery of whole-limb length.

While peripheral nerve injury resulted in an immediate and significant change in whole-limb length, the observed deficits in limb orientation were much smaller. We expected a smaller effect on orientation, which is determined largely by the swing phase of gait and powered by flexor muscles which were uninjured in our study. Chang et al. (2009) also showed that geometric constraints can make whole-limb orientation less sensitive to kinematic deviations of the distal limb segments compared to whole-limb length. Despite their small magnitude, the changes in limb orientation exceeded one standard deviation of the pre-injury mean across most of their stance phase. Thus, we conclude that like whole-limb length orientation was not immediately conserved, meaning that its correction, if necessary, would require adaptation.

### Hierarchical nature of locomotor recovery

Recovery of whole-limb kinematics was achieved after localized muscle paralysis by utilizing a new combination of component joint kinematics. Whole-limb length returned to normal through a two-step process involving immediate increases in extension primarily at the hip joint and variously at the knee joint, followed by a gradual yet incomplete return of subnormal extension at the ankle and MTP joints. Coordination across joints in recovery was clearly demonstrated by our observation that progressive reduction in ankle yield discontinued once whole-limb kinematics reached normal values. The arrest of ankle recovery that occurs when whole-limb kinematics reach full recovery suggests locomotor compensation after injury may be controlled through a hierarchical control structure (Loeb et al., 1999), where global task-level parameters such as whole-limb kinematics are prioritized over individual joint function. Notably, the coordinated changes in joint kinematics appear to be guided by a residual representation of pre-injury whole-limb kinematics acting as the functional goal of this adaptation. There is evidence for sensorimotor representations of limb-level parameters in the cat central nervous system (Bosco et al., 2006; Drew et al., 2002, 2008; McVea et al., 2009; Ting and Macpherson, 2005). Bauman and Chang (2013) observed that rats also respond to peripheral nerve injury by preserving limb kinematics as joint kinematics deficits lingered. Similar findings in healthy human locomotion further indicate that joint dynamics are coordinated on a step-by-step basis to stabilize limb dynamics (Auyang and Chang, 2013; Yen et al., 2009), suggesting that hierarchical control of the limbs may be routinely used during legged locomotion. Taken together, locomotor compensation by way of substituting alternative kinematics solutions may provide an advantage over full neuromuscular restitution, i.e. nerve regeneration and muscle reinnervation. Substitution provides faster functional recovery compared to restitution, and may ultimately establish a ‘good enough’ locomotor strategy that prevails long after injury.

Although whole-limb kinematics may function as control targets of the central nervous system, other targets, such as limb force generated against the ground, may be equally pertinent. Humans are suggested to control limb force during locomotion (Auyang and Chang, 2013; Selgrade and Chang, 2015; Toney and Chang, 2013, 2016; Yen et al., 2009; Yen and Chang, 2010). Recent work in guinea fowl indicate that running birds may be more likely to use limb angle and limb loading as control targets (Blum et al., 2014; Daley and Biewener, 2006). As it is difficult to differentiate limb force from length representation in the cat central nervous system (Bosco et al., 2006), further study is needed to distinguish subtleties that may exist across species and locomotor gaits, in addition to differentiating between discrete locomotor responses to obstacle avoidance versus chronic neuromuscular injury.

### Limitations to interpretation

Kinematic data are unavailable, either in this or previous reports to our knowledge, for cats sham controlled for nerve-injury surgery. We cannot rule out, therefore, the possibility that factors associated with the surgical procedure for cutting nerves influenced whole-limb recovery, which was first measured 20 h after surgery. Among factors potentially confounding recovery from nerve injury are surgical anesthesia and post-operative pain or discomfort at the surgical site. We ascribe minimal concern to anesthesia, since the isofluorane clearance from all tissues occurs in less than four hours (Schmidt and Dudziak, 1984). Pain at the injury site is more difficult to rule out, although it would certainly be a relevant factor in a realistic assessment of the nervous system's ability to recover normal function. In our studies, pain and discomfort were treated immediately postoperative with buprenorphine and for 5 days thereafter with an oral NSAID (see Materials and Methods). The potential for these agents to affect kinematics is difficult to assess, although we found no systematic change in treadmill stride length over the course of study.

In summary, our longitudinal study of the injury response from four individual cats documents the unexpected finding that whole-limb kinematics is not immediately conserved following localized nerve injury, regardless of affected muscles. These results provide important insight into what appears to be a progressive neuromuscular process of locomotor compensation. Our observations showing preferential recovery of limb kinematics over individual joint kinematics provide support for a hierarchical control strategy of compensation in legged locomotion.

## MATERIALS AND METHODS

### Study design

Kinematics data collected from cats engaged in separate studies of nerve injury in our lab made it possible to address this objective with existing data. Limb kinematics during treadmill walking were available for four adult cats studied over several days beginning soon after nerve injuries that paralyzed primary ankle extensor muscles to produce similar losses of ankle joint extensor torque. These features, described below in detail, met our study objective through a case study of longitudinal data from four individual animals.

### Animal care

All procedures were performed according to protocols approved by the Wright State University Laboratory Animal Care and Use Committee. Four purpose-bred (Class A) adult cats (mass 2.5–4.5 kg), two male and two female, were acquired from Liberty Research, Inc. (Waverly, USA) and housed separately by gender in large open-floor rooms (35 m^2^) where they had ample space to walk, run, climb and play. Ambient temperature (24°C) and light-dark cycles (12 h) were controlled and dry food and water were provided *ad libitum*. All cats were studied before and after nerve injury as described below.

### Survival surgery

All four cats were subjected to a survival surgery in which selected ankle extensor muscles were denervated in the left hindlimb. Surgery was performed following intramuscular injection of pre-anesthetics (ketamine 10 mg/kg and xylazine 1 mg/kg, Med-Vet International, Mettawa, USA), and tracheal intubation was used to deliver gaseous anesthesia (1.2-2.5% isoflurane in 100% oxygen) throughout surgery, and intravenous (IV) catheterization of the right cephalic vein for administration of fluid (lactated ringer; 10 ml/h.). Oxygen saturation, pulse rate, respiration rate, end-tidal CO_2_ and body temperature were monitored throughout surgery, typically completed within 1.5 h.

The nerves supplying the medial gastrocnemius (MG), lateral gastrocnemius (LG), soleus (S) and plantaris (P) muscles were exposed through an incision (∼4 cm) overlying the popliteal fossa in the left leg. Transection of nerves to these muscles produced robust changes in ankle joint kinematics trajectory as described in earlier studies (Carrier et al., 1997; Chang et al., 2009; Maas et al., 2007; Pearson et al., 1999). In two cats, the nerves supplying the MG and LG muscles were isolated, severed by scissors, and immediately rejoined end-to-end (MG to MG; LGS to LGS) by 3-4 ties of non-absorbable monofilament suture (10-0 Ethicon) passed through the epineuria. For these two cats, identified as xG1 and xG2, kinematics study was performed up to 3 weeks after injury, during which time there was little or no significant functional reinnervation of muscle (Gordon and Stein, 1982; Gregor et al., 2018). In the other two cats, designated xLGSP1 or xLGSP2, the nerves supplying LG, S and P were severed and not repaired, but were instead ligated with suture on both proximal and distal cut ends to prevent any muscle reinnervation. In all cases, complete denervation of selected muscles was verified during surgery when electrical stimulation of the corresponding nerve, applied by bipolar electrodes (A-M Systems, Sequim, USA) proximal to nerve injury, failed to evoke muscle contraction. Robust contraction of the remaining ankle extensor muscles, elicited by electrical stimulation of the tibial nerve proximal to the surgical sites, demonstrated that these intact muscles maintained functional innervation. When surgical treatment of nerves was complete, incisions through fascia and skin were closed in layers by 4-0 Vicryl-plus absorbable suture, and anesthesia was discontinued after administration of analgesic (buprenorphine, 0.1 mg/kg SQ, Sigma-Aldrich) and antibiotic (Enrofloxacin 5 mg/kg IM, Sigma-Aldrich). Cats were transferred to a recovery cage where they were kept comfortable and warm, and were continuously monitored until they regained full consciousness and standing posture, consistently within 3 h. An antibiotic and a NSAID (Metacam0.2 mg/kg, Sigma-Aldrich) were given post-operatively for 3 to 5 days following surgery.

The complete set of muscles acting to produce ankle extension in normal cats includes MG, LG, S, P and the flexor hallucis- and digitorum-longus (FHL and FDL). For each of these muscles in the adult cat, Lawrence and Nichols (1999) report maximal ankle torque produced at an ankle-joint angle of 110°. From these values, we estimate that for each of the two kinds of injury studied here, (xG and xLGSP), the total maximum ankle-joint torque produced in the sagittal plane by all injury-spared muscles combined was reduced to approximately 40% of normal (∼70/160 N-cm).

### Kinematics of treadmill walking

Before surgery, all cats were trained by food reward to walk within a clear plastic enclosure (120 cm×120 cm×60 cm) mounted over the moving belt of a motorized treadmill (Vision Fitness Treadmill T9600 Wisconsin, USA). Kinematic measurements began when cats were trained to the point of completing at least 20 contiguous steps at the constant speed of 0.7 m/s, which was the speed most readily adopted by all four cats. Kinematic data were collected longitudinally for each of the four cats, beginning at least 1 week before nerve injury, and extending 5 days or longer afterwards (see Results). Data were collected up to the day before surgery (pre-injury control) and resumed within 20 h post-surgery, with each session lasting less than 1 hour, once per day. Without exception, cats gave no evidence of discomfort or disinterest in treadmill walking or food reward following nerve injury. Note that cats were housed in the recovery cage for the first 5 days after surgery to permit recovery of the skin incision before being returned to the colony room. The first recording session captured each cat's very first treadmill steps following surgery.

For purposes of filming limb kinematics, colored-tape markers were temporarily placed on the skin at the superior iliac crest (IC), greater trochanter (hip), lateral malleolus of the fibula (ankle), fifth metatarsal-phalangeal joint (MTP) and the distal-most part of the fifth digit (toe) on the left leg (Fig. 1). Treadmill locomotion was recorded using a motion capture system (KineTracer; Kissei Comtec, Nagano Prefecture, Japan), utilizing two cameras (DragonFly2; Point Grey Research, Richmond, British Columbia, Canada) that filmed at 30 frames p/s. Filming began once the treadmill achieved a fixed speed, and cats maintained steady speed typically for 30 s and about 20 steps in each filming trial. Films from three to five trials were recorded in each daily session and then digitized and stored on computer.

The spatial location of the markers was processed and analyzed offline using KineTracer software (Kissei), which allowed for assessment of hip, knee, ankle and MTP joint angles; i.e. the joint kinematics. Since skin movement errors are very high for the knee, its position was triangulated using the measured lengths of the femur and tibia (obtained post-mortem) and the positions of the hip and ankle markers (Chang et al., 2009). Once the knee position was calculated, it enabled assessment of the included angles of hip, knee and ankle joints. Whole-limb kinematics were also calculated, including limb length and limb orientation (Fig. 1). Limb length was defined as the straight-line distance between the markers on the iliac crest and toe, and limb orientation as the angle between the ground and the line defining limb length (Chang et al., 2009). Paw contact (PC) to paw off (PO) were assessed using KineAnalyzer software as, respectively, the time of maximum and minimum velocity of the toe marker in the x-axis (rostral/caudal axis). These estimates of PO and PC were then visually verified and, when error was evident, corrected using KineAnalyzer. Although error is expected for measurements of PC and PO made from foot velocity, the error is not systematic and is not affected by time after nerve injury (Pantall et al., 2012).

### Statistical analyses

Whole-limb and individual joint kinematic data collected from multiple steps per session of treadmill walking met criteria for using means and standard deviations as valid descriptive statistics. Statistical comparisons were made within individual cats from data obtained at different time points before and after nerve injury.
